# Should North America’s first and only supervised injection facility (InSite) be expanded in British Columbia, Canada?

**DOI:** 10.1186/1477-7517-10-1

**Published:** 2013-02-16

**Authors:** Ehsan Jozaghi, Martin MA Andresen

**Affiliations:** 1 School of Criminology, Simon Fraser University, 8888 University Drive, B.C. V5A 1S6, Burnaby, Canada

**Keywords:** Supervised injection facility, Harm reduction, Drug policy

## Abstract

**Background:**

This article reports qualitative findings from a sample of 31 purposively chosen injection drug users (IDUs) from Vancouver, Surrey and Victoria, British Columbia interviewed to examine the context of safe injection site in transforming their lives. Further, the purpose is to determine whether the first and only Supervised injection facility (SIF) in North America, InSite, needs to be expanded to other cities.

**Methods:**

Semi-structured qualitative interviews were conducted in a classical anthropological strategy of conversational format as drug users were actively involved in their routine activities. Purposive sampling combined with snowball sampling techniques was employed to recruit the participants. Audio recorded interviews were transcribed verbatim and analyzed thematically using NVivo 9 software.

**Results:**

Attending InSite has numerous positive effects on the lives of IDUs including: saving lives, reducing HIV and HCV risk behavior, decreasing injection in public, reducing public syringe disposal, reducing use of various medical resources and increasing access to nursing and other primary health services.

**Conclusions:**

There is an urgent need to expand the current facility to cities where injection drug use is prevalent to reduce overdose deaths, reduce needle sharing, reduce hospital emergency care, and increase safety. In addition, InSite’s positive changes have contributed to a cultural transformation in drug use within the Downtown Eastside and neighboring communities.

## Background

People infected today with HIV/AIDS are increasingly intravenous drug users (IDUs) who are involved in sharing injection equipment [1]. In Canada for example, one in four new cases of HIV is attributed to sharing needles [2]. The situation has been particularly bad in Vancouver, Canada with one of the highest outbreaks of HIV in the developed world [3]. In addition to the spread of infectious diseases, British Columbia, Canada has a drug overdose epidemic, with up to one death per day being documented in recent years [3,4].

Though mortality (overdose) and blood borne pathogens epidemics (such as HIV and HCV) are centered in the Downtown Eastside of Vancouver, they are national and provincial problems requiring immediate action. Conservative estimates suggest that there are now more than 125,000 people who inject illicit drugs in Canada [5,6]. In British Columbia, it is estimated that there are approximately 20,000 injection drug users whose lives are further marked by extreme poverty, mental illness and homelessness [7-9].

In order to reduce the community, public health and fiscal impacts of injection drug use, North America’s first and only supervised injection facility (SIF), known as ‘InSite’, opened its doors September 22, 2003 in Vancouver’s Downtown Eastside [7]. To date, there have been 1.5 million visits to InSite with 700 to 800 injections per day [10-13]. The first several years of evaluation have yielded an array of scientific output, including more than 30 peer-reviewed studies. These publications indicate that InSite provides a range of benefits to its clients and society.

Therefore, if North America’s only supervised injection facility has numerous positive benefits (operating at full capacity with potentially a few thousand injection drug users who reside in the vicinity of the facility alone), the question of whether or not the program should be expanded is topical [14]. In fact, HIV, HCV infections and illicit drug overdose deaths are documented in virtually all settings in British Columbia, Canada where injection drug use is prevalent [15]. Furthermore, improving access to, and availability of supervised injection through expansion may help reduce persistent risk behaviour among IDUs [16,17]. As a result, this study explores the potential of expanding InSite to more locations throughout British Columbia. In addition, this study explores the current status of injection drug users who reside in cities that have no access to supervised injection facilities such as, Surrey and Victoria, British Columbia.

## Methods

Beginning in October 2009, participants living in Surrey, Vancouver and Victoria, who had injected illicit drugs in the previous month were recruited to participate in the study. The participants were eligible for the study if they had injected illicit drugs at least once in the previous month, were 19 years or older and provided informed oral consent. They received CAD$10 reimbursement for their participation at the end of a semi-structured interview. The study was approved by Simon Fraser University’s Research Ethics Board.

**Figure 1:**
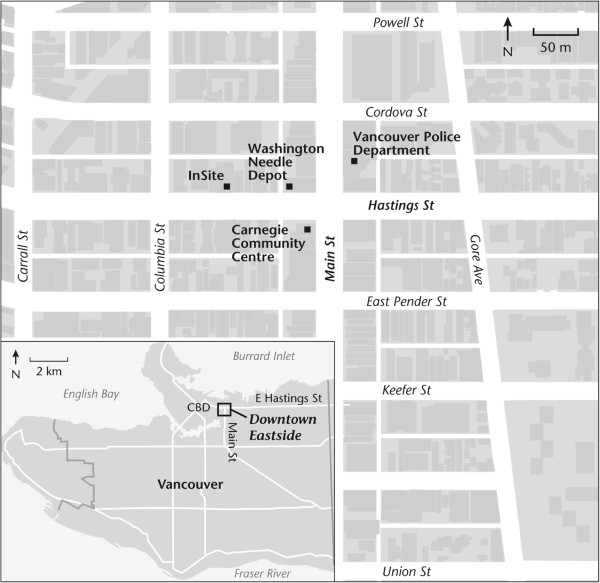
Map of the Downtown Eastside of Vancouver.

The city of Vancouver’ Downtown Eastside neighborhood was chosen as one of the recruitment locations because it is home to North America’s only supervised injection facility (see Figure 1).

**Figure 2:**
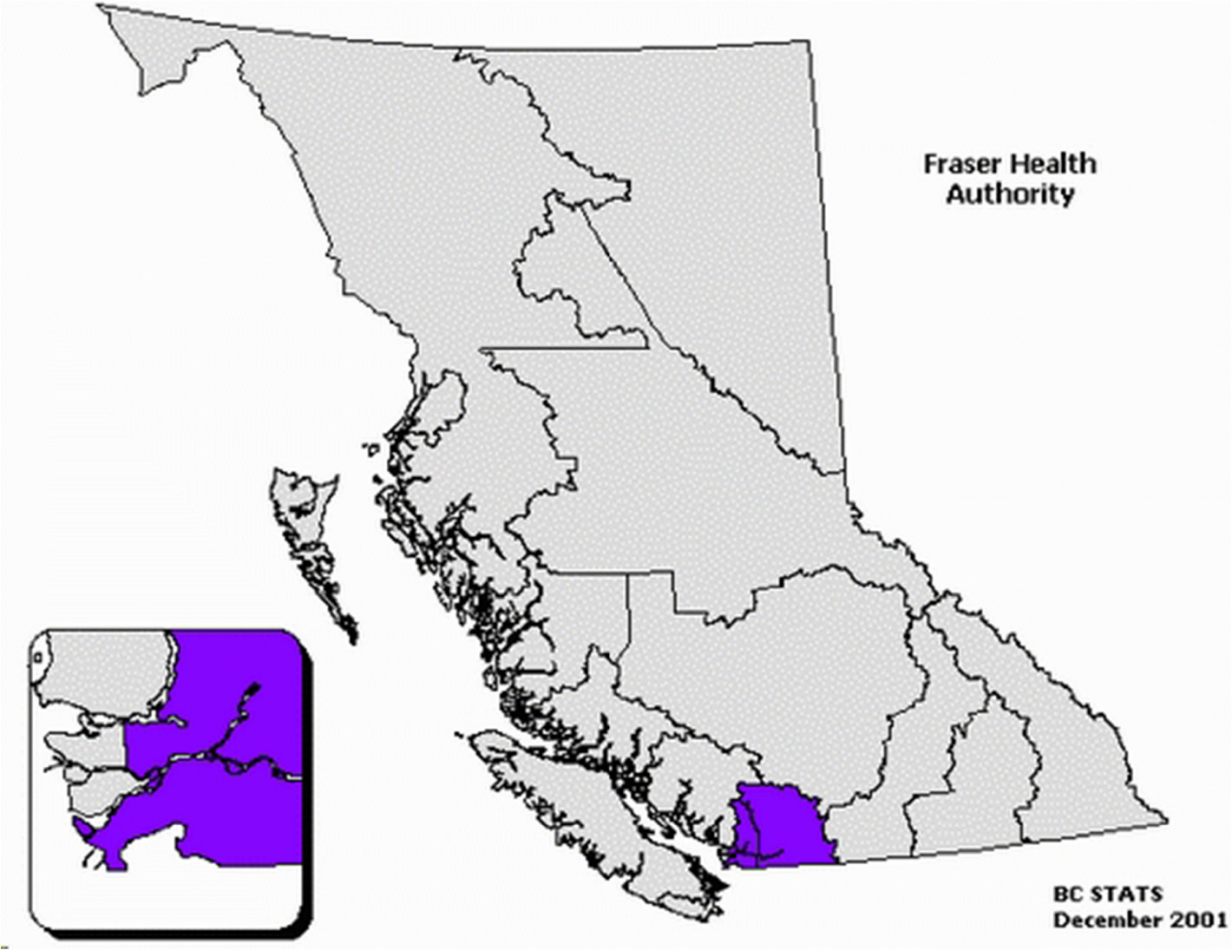
Fraser health location within the Province of British Columbia.

The city of Surrey’s Whalley/City Centre neighborhood was chosen as another recruitment location because the neighborhood is home to a needle exchange depot, a health center and a homeless shelter that attract a large number of IDUs. It is also estimated that the Fraser health authority region that includes Surrey has the second highest population of IDUs in BC with approximately 16,000 [18] (See Figure 2).

The city’s proximity to Vancouver’s DTES is unique because many IDUs can access InSite by travelling on the train for 45–60 minutes. Finally, Victoria, is a small city located on the southern tip of Vancouver Island. The city’s Downtown neighborhood was chosen as a recruitment location because the city’s fixed needle exchange depot was recently forced to shut down.

In Vancouver, 16 interview participants were recruited through key informants in the city’s Downtown Eastside neighborhood. The key informants helped to establish a rapport and trust among IDUs. In order to maintain confidentiality, all names used in this paper are pseudonyms. The key informants also proved to be instrumental in guiding the sampling selection based on participants’ drug of choice, years of injection, ethnicity and gender as outlined in Table 1.

**Table 1:** Characteristics of the sample of IDUs of Vancouver

**Name**	**Gender**	**Age**	**Ethnicity**	**Drug of choice**	**Years of injection**	**Medical condition**	**Year selected**
Tania	Woman	46	White	Heroin & Crack	27	HCV & HIV	2011
Joe	Man	60	White	Heroin	45	HCV	2011
Maxim	Man	50	White	Heroin & Crack	35	HCV & HIV	2011
Michelle	Woman	30	White	Heroin & Crack	12	HCV	2011
Ashley	Woman	43	First Nation	Cocaine, Heroin & Crack	20	HCV	2011
Martin	Man	46	White	Cocaine & Heroin	10	Bipolar Disorder	2011
Jack	Man	46	First Nation	Heroin & Crack	30	HCV & HIV	2011
Catherine	Woman	53	White	Heroin & Crack	23	HCV	2011
George	Man	53	White	Cocaine & Heroin	2	HCV & Diabetic	2011
Sam	Man	37	First Nation	Heroin	10	HCV & Abscesses	2011
Alex	Male	47	Caucasian	Heroin & Crack	30	HCV	2009
Ayatollah	Male	50	Middle Eastern	Heroin	20	HCV	2009
Dan	Male	37	First Nation	Heroin	10	HCV & Abscesses	2009
Lisa	Female	39	First Nation	Cocaine & Crack	15	Bipolar Disorder	2009
Niki	Female	30	Caucasian	Cocaine & Crack	14	HCV & HIV	2009
Shane	Male	29	Caucasian	Heroin	13	HCV & HIV	2009

In Surrey, nine participants were recruited in areas where most IDUs congregate. Snowball sampling proved to be instrumental in guiding the selection process. There were no refusals of invitations to participate in the study. All the participants were only interviewed once. Based on Table 2, a variety of participants were selected.

**Table 2:** Characteristics of the sample of IDUs of Surrey

**Name**	**Gender**	**Age**	**Ethnicity**	**Drug of choice**	**Years of injection**	**Medical condition**	**Year selected**
Cindy	Woman	42	White	Cocaine & Crack	18	Nil	2011
Gary	Man	56	White	Cocaine & Heroin	44	HCV & Abscesses	2011
Brian	Man	47	White	Cocaine & Crack	17	HCV & HIV	2011
Scott	Man	42	White	Cocaine & Heroin	30	HCV	2011
Chris	Man	49	White	Speed & Heroin	15	HCV	2011
Daniel	Man	41	First Nation	Cocaine & Heroin	15	HCV	2011
Holly	Woman	50	White	Speed & Crack	20	Bipolar & Cancer	2011
Jenny	Woman	47	White	Cocaine & Heroin	30	HCV	2011
Kayleigh	Woman	28	White	Heroin & Crack	10	HCV	2011

In Victoria, six participants were recruited in neighborhoods where most IDUs are known to congregate. Recruitment of participants was facilitated by a key informant (Table 3).

**Table 3:** Characteristics of the sample of IDUs of Victoria

**Name**	**Gender**	**Age**	**Ethnicity**	**Drug of choice**	**Years of injection**	**Medical condition**	**Year selected**
Henri	Man	50	White	Heroin & Crack	33	HCV, HIV, Cancer & Diabetes	2011
Fraser	Man	49	White	Heroin & Crack	35	HCV & HIV	2011
Kila	Woman	30	White	Cocaine, & Heroin	10	Abscesses & MRSA	2011
Loren	Woman	29	White	Heroin & Meth	12	HCV	2011
Melanie	Woman	44	White	Heroin	2	Bipolar & HCV	2011
Thomas	Man	35	White	Heroin & Meth	18	HCV	2011

The open ended, semi-structured interviews were facilitated through the use of an interview guide. The interview guide encouraged discussion about SIF, the impact of SIF upon their behavior, and elicited suggestions related to the ways it can be improved. The themes followed throughout the interview in Vancouver were along the following four dimensions: 1-experience prior to opening of InSite, 2-experince after opening of InSite, 3-changes that they have noticed in their behavior and 4-an open discussion about anything raised during the interview. The themes followed in Surrey and Victoria was along the following three paradigms: 1-experience on the street 2-what is the difference between injecting at InSite or on the street (if they have attended a SIF) 3-should InSite be expanded in their community. To reduce distortion of data due to social desirability responses, interviews were conducted in a conversational format as drug users were actively involved in their routine activities. This classical anthropological strategy of participants’ observation allowed a triangulation of responses to conversational prompts [19].

The qualitative data were reviewed, and all text segments were subsequently subjected to a thematic analysis using NVivo 9 software. Initially an open coding method of searching for similar words or repeating phrases was employed. Twenty coding categories emerged. Researchers warn of the tendency for coding schemes to become powerful conceptual grids from which it is difficult to escape [20]. Therefore, each coding category was reviewed again at a later date, this time using the key themes as coding categories. Each coding category was reviewed independently for latent meanings and common ideas. The main thematic analysis focused on the social processes and experiences of injecting on the street.

Validity is an important concept in both quantitative and qualitative research that was considered in this analysis. Validity is defined as, “truth: interpreted as the extent to which an account accurately represents the social phenomena to which it refers” [21]. In order to maintain validity in this research and avoid “anecdotalism”^a^, quotes were considered both in the context of the interview and as standalone representations of a theme.

## Results

### Overdose

The most common narrative offered by the study participants—who have used InSite—was that InSite is saving lives. In fact, most participants such as George, can recall the dire situation of the Downtown Eastside prior to the opening of North America’s first supervised injection site:

*After they opened InSite, It was like a warm hug from God … I mean people used to die here from overdose almost every day … Almost everyday people were hauled out of an alley, behind dumpsters by paramedics after they went blue.*^*b*^

This notion of fear and death associated with overdose when injecting outside reinforces the safety and security that many participants have come to associate with InSite. In fact, all the participants who have used InSite have seen an overdose or have experienced an overdose at InSite and all of them agree that InSite has reduced overdose deaths.

The notion that InSite saves lives is echoed by other users who have seen a reduction in overdose in the allies in the vicinity of InSite because most IDUs prefer to come to InSite. In effect, IDUs who used InSite have come to associate outside injection with a substantial risk of death that they are simply not willing to take. As a result of InSite, there are fewer public injections. InSite has also reduced public syringe disposal and substantially reduced the use of various medical resources such as ambulances and hospital emergency care. In essence, according to Joe:

If it wasn’t for InSite you would see 150 people sitting down in the alley with rigs [needles] sticking out of their arms, flagging blood in their needle, Y’know, ODing left and right every day and leaving their rigs around … They needed ambulances up in the alleys constantly … Today you rarely see people fixing outside, especially in and around InSite.

However, in cities that have no access to a safe injection site, such as Surrey, regular overdose death is the reality. All the participants in Surrey have known a person who has died of overdose. For example according to Kayleigh:

I know of at least three to four people a year that I knew personally that ODed and eventually died as a result. You see ambulances coming to the front room [homeless shelter] all the time. At least twice a week people are ODing down here.

In most cases, an overdose in both Surrey and Victoria is accompanied by death. IDUs do not have the knowledge or expertise to help someone in an overdose case. Further, IDUs don’t have access to a cell phone or a public phone to call 911.

### Sharing

Not having access to a supervised injection facility can do more damage than a simple overdose; it can help to spread infectious diseases such as HIV and HCV. According to Ashley, “before InSite, people would’ve been fixing everywhere in public. HIV and Hep C was everywhere ‘cause junkies where sharing rigs or didn’t have access to clean ones”. But the opening of North America’s first supervised injection site has changed sharing behavior and public injection scenes in the Downtown Eastside of Vancouver where IDUs would not share again or inject outside. Moreover, because they perform all of their injections at InSite, they curb the spread of infectious diseases like HIV/HCV and injection related illnesses such as abscesses. Furthermore, the participants report that the provision of sterile syringes, the ancillary injecting equipment and safer injecting advice by nurses serve to reinforce the permanent adoption of safer injecting practices. As Maxim explained,

Because of InSite, I don’t like to do it outside anymore, I don’t want people seeing me fixing … But before InSite we were fixing in the shooting galleries. It was so unhealthy, Y’know, I ended up with HIV because of the area. And now the only reason I come to InSite is to slow down the spread. Not only that, InSite is such a clean experience … You don’t have to use puddle water for injection. There are nurses on staff there that have taught us about diseases and shit like that we’d be scared not to use a clean needle … Also, I have taken upon myself to give shit to junkies who are fixing outside, I usually tell them: have little respect for people for God’s sake. We have a place, why don’t you do it at InSite. And I’ve convinced few people to do it at InSite.

This dramatic advocacy for InSite and on behalf of other users by participants who once injected and shared outside is something unique to the Downtown Eastside community. Unfortunately, no such advocacy or health consciousness was observed in Victoria or Surrey. In fact, sharing needles is still prevalent in cities that don’t have access to a supervised injection site. For example, as Gary explained:

I have seen people picking needles off the ground and using them. My wife picked one up down here couple of years ago and she wanted to return it to the needle exchange depot so she could get a credit. The rig was full of blood, and this junkie bug her so he could try what was in there. He didn’t know what was in there or who has used it, but he wanted that rig so bad so he could get high.

In effect, sharing behaviors within the IDU population is an established factor that is thought to lead to a substantially higher risk of HIV infection, even if practiced relatively infrequently [22]. Participants in Surrey and Victoria attribute sharing and reusing needles to inaccessibility of needles in both cities. In fact, closing the recent needle exchange depot in Victoria has resulted in more sharing within the IDU population.

### Safety

In addition to the improved changes in behavior and shared health concerns described above, InSite has helped to bring safety and security to participants who use the facility. Before opening of InSite, according to Catherine, fixing outside was accompanied by various risks including the risk of theft:

When I was going to the alley to do my fix, I got robbed so many times. For example, you do your fix, and somebody takes off with your purse, Y’know. Hands are busy and you can’t run after them. I lost my welfare cheque more than once … At least at InSite you know you’re not getting robbed.

In addition to risk of theft, participants who have used InSite have come to associate outside injection with significant risk of bodily harm or even death. As a result, most of those who describe injecting at InSite are not willing to inject outside. In other words, once safer injecting habits and feelings of safety are established within InSite, it becomes more likely that IDUs will come to InSite for every injection, reducing the risk of sharing or getting attacked. For instance, according to Martin:

Junkies would do anything for the money, they will fucking stab you for it. That’s why fixing outside is not safe. I saw somebody getting stabbed in one of these alleys few years ago. He was trying to fix, then somebody jump on him, trying to steal his bag. The poor guy tried to fight the mob and they fucking killed him. They stabbed him in the neck. No fucking lie, I think about that every single day, that’s why I always try to fix at InSite.

Furthermore, IDUs who come to InSite escape police arrest because they will not be questioned by police for having an illegal substance. Injecting in public brings a significant risk of arrest and questioning by police if they are caught in the act. According to Joe, InSite has become a refugee camp for IDUs of the Downtown Eastside who want to escape, disease, theft, arrest and death:

First of all you’ve got a clean, safe place; nobody is gonna bother you or you don’t have people trying to steal from you. You don’t have police coming and hassling you … That’s why people are always hiding from cops and fixing in washrooms or behind dumpsters. But then you’re facing over dose ‘cause you might do a bigger whack. But InSite is such a stress free, cop free, disease free, OD free environment that I call it the refugee camp for junkies.

Although InSite mitigates the risk of violence and arrest for IDUs who are using the facility and many may describe it as a ‘refugee camp for junkies’, the daily reality for IDUs who don’t have access to the site is formidable. IDUs that live in Surrey, according Scott, have to endure risk of violence and theft everyday when they fix:

Everyday there is a few fights. You can bump into the wrong person and have three guys jump on you and rob you … A lot of girls get robbed. They just walk up and take their money when they have the chance, and the best fucking time to rob someone is when their fixing.

In addition to the risk of violence and theft that seems to be the daily reality of street life for many IDUs who don’t have access to InSite, according to the participants, the risk of police arrest is another factor in their daily lives.

### Services

Participants’ accounts indicate that availability of services and equipment at InSite has made a huge difference in their lives. In effect, according Tania, accessibility of injection equipment and ancillary services provided at InSite reduces sharing behavior in the vicinity of InSite:

There used be a lot of sharing down here before InSite. Today, you don’t see that anymore. People seem to understand the risk. There are enough clean rigs going around. I used to see people using water from drain pipes and things. But at InSite you can get all your supplies.

In addition to accessibility of injection equipment, ancillary services, and available nurses, counselors and staff, InSite helps transform the public injection scene of the greater Vancouver area. This is particularly true for those who are the most marginalized, such as Maxim:

The staff are so helpful, anything you need, all you have to do is ask, if you need housing, or you need to get off the street, Y’know. For example, when I was first diagnosed at VGH [Vancouver General Hospital], the Dr said: you have Hep C and you’re HIV positive and he walked out of the room. I wasn’t told where to go … It wasn’t till I came to InSite for my injection that one of the counselors told me about going to St. Paul’s and he set up the appointment … The staffs genuinely do care about us.

Moreover, participants’ accounts indicate that staff and nurses at InSite gain awareness holistic strategies and approaches that go beyond simply providing care. In effect, the staff has been able to create dignified, caring and trusting bonds that build foundations for change through personal empowerment. According to Ashley:

I’ve had the chance to talk to nurses, in fact, I had a skin rash … and they changed my bandages … they also paid for my transportation so I could see a doctor … Also when my son died I was really hurting and I was gonna OD myself, and when I got there, I talked to one of the staff and they gave me hope to stay alive … Just because of the programs at InSite, my drug use is now the third of what it was. They gave me positive thinking and stuff, and I realized I can do it.

The relationship that exists between the staff and IDUs at InSite facilitated more than 2,000 referrals to addiction services, with 800 of these referrals to addiction counseling [23]. Furthermore, the services provided by nurses at InSite, such as changing bandages for bites or abscesses reduces emergency care utilization significantly. However, IDUs in municipalities with no supervised injection site are having difficulty meeting their most basic need: finding a clean needle. In effect, in both Surrey and Victoria the most common narrative was associated with inaccessibility of clean needles. According to Daniel:

It’s hard to get a needle down here, [the needle depot] closes at six o’clock and they don’t open till noon. So a lot of people go without a clean one. And that happens all the time. … I remember … this fellow asking me if I had a syringe. I looked in my bag: All I had was a used one. And I told him that I don’t want to sell you a used one … But he still insisted … So I told him again that I don’t have bleach and I have Hep C. But … he didn’t care. He bought the syringe.

Clean needle accessibility is a major problem in Victoria with the number of clean needles distributed in Victoria falling by 15,000 per month since the closure of the needle exchange office in the Downtown Victoria [24].

### Changes in behavior

Those IDUs who use InSite have come to associate InSite as their ‘community center’. Many feel right at home at InSite because staff and nurses are non-judgmental and respectful toward everyone who uses the facility. InSite is a place where all IDUs gather for support and acknowledgment. For example, as Sam describes:

InSite has helped junkies to feel a sense of belonging, I call it the community center for junkies ‘cause we are welcomed there, we can stay in for a coffee or juice, see our buddies, watch TV in the chill room or talk to counselors. We are not judged for who we are, or what we do. Staff gives us respect and they don’t judge us. At InSite we actually feel like that we exists.

The influence of InSite goes beyond changing sharing behavior and reducing overdose death, enhancing safety or enhancing a positive image within IDUs. Services provided by nurses and staff at InSite inspire many IDUs to become safety and educational ambassadors within their own community. According to Sam:

I always carry extra rigs in my pocket to give out to other junkies. We try to promote InSite at every chance we get … If we see somebody new in town, we try to take him to InSite. We are tired of seeing people OD in alleys; we are tired of seeing rigs on the ground. I also go around in alleys and pick up rigs and bring em back to InSite or the needle depot.

Furthermore, participants who have been coming to InSite for a few years felt empowered to help others. Many of them had seen the transformative power of InSite (either through counseling, social support, or overdose emergency care) and craved for change within their own community. This empowering change is even observed in people who travelled from surrounding municipalities such as Surrey. For example, according to Brian:

I travel to InSite at least twice a week … Every time I come here I grab few boxes of needles, water, alcohol wipes to take it to Surrey. I give those out to other junkies. It’s harsh when you need a rig and you can’t find one. The needle depot in here has limited hours … I’ve also told about InSite to few people.

Their new roles as a result of self-empowerment have the potential to mediate patterns of infectious disease and mortality, and eventually change lives amongst the most marginalized IDUs.

### Access

The most common problem associated with InSite according to participants is related to the lineup and access to the site. In essence, participants believe that the 12 booths that are currently in operation should be expanded so fewer users would have to substitute InSite for the alleys. InSite seems to be inaccessible during welfare week in particular when IDUs are issued their disability or social assistance cheques. During that week, the only alternative for many IDUs is to use the alleys that involve risking arrest, theft, violence or overdose. If they are not homeless, they still risk overdose if they inject at their single occupancy units. This is particularly true according to Martin, if they are ‘dope sick’:

There are times when there is long waiting list in there, such as welfare week. You basically have 60 people ahead of you … Long waiting list at InSite isn’t like waiting for a hockey game, but when you’re waiting to put a needle in your arm and you haven’t had a hit for a day and a half, even five minutes is too fucking long.

The underlying message that InSite needs to be expanded is echoed by other users who believe that inaccessibility is acutely felt during morning hours when InSite is closed. IDUs who live in Surrey and Victoria indicate they would use a supervised injection facility if such a site ever opened. Many participants stated they would use a supervised injection facility for safety reasons, others emphasized the need to avoid hazards of the street, while many stated they would use a supervised injection facility to stay alive. For instance, according to Jenny:

I know for a fact that if they open an InSite, a lot people would go and use it. Right now people are fixing in alleys, crack shacks or drug houses … Also, if you OD nobody is gonna care … they’ll take you and throw you outside. That’s … why an injection site would be good ‘cause there would be people there that can help you. You would feel safe in there. There is no risk of … sharing.

## Discussion

The present study was conducted on the premise of assessing the transformative role of InSite in the lives of IDUs. In addition, this study explored the current status of injection drug users who reside in cities that have no access to supervised injection facilities such as, Surrey and Victoria, British Columbia. The ultimate objective of this study was to determine whether the current supervised injection facility needs to be expanded to other cities. The results reveal a positive change in many aspects of IDUs who are increasingly relying on the services offered at InSite. In fact, the findings of the present study suggest that InSite prevents drug overdose deaths and reduces overdose deaths in surrounding areas. InSite has also reduced HIV and HCV risk behavior (e.g., sharing needles), decreases injection in public, reduces public syringe disposal and substantially reduces use of various medical resources such as ambulances and hospital emergency care. In addition, InSite has increased access to nursing and other primary health services crucial for curbing the spread of infectious disease and injection-related illnesses.

Aside from the numerous positive accounts of InSite reinforced by peer reviewed studies, the current study reports four new findings not previously discussed. First, InSite has created a ‘refugee camp’ for IDUs by allowing them to escape the theft, violence, and murder they would normally face on the streets. Furthermore, IDUs who come to InSite escape police arrest and questioning. The new sense of safety that many IDUs have come to associate with InSite reinforces their reliance on the facility for all their injection needs.

Second, the most prominent finding in this paper is related to the significant transformation in IDUs’ roles and behaviors. InSite’s positive changes mentioned above (such as not sharing, improved health, less overdose death, plus changes in enhanced safety, helping others and collective identity) have contributed to a cultural transformation in drug use within the Downtown Eastside and neighboring communities. Those who increasingly rely on InSite have gradually become active within their community, trying to alleviate misery and improve lives in the Downtown Eastside. This paper identifies participants who strive to better their peers’ health and their communities’ self image as educational and safety ambassadors.

Third, there is a need to expand the program in the Downtown Eastside of Vancouver to reduce the waiting time. This finding is not surprising because it is estimated that the pilot program with only 12 injections seats is located in a neighborhood that contains 5000 IDUs [17]. A similar study also suggests waiting times and travel distance to the facility as significant barriers to InSite use [25]. Finally, results in this study depict the lives, stories and circumstances of IDUs who live in municipalities that do not have access to a supervised injection facility. InSite is the only supervised injec-tion facility in North America, so their stories and circumstances have relevance to other Canadian and American cities. Based on the results, IDUs in such cities are faced by over dose death, disease, violence, theft and arrest on a daily basis. As a result, there is an urgent need to open similar supervised injection facilities in cities with significant IDU populations. Further, the results suggest that a high proportion of IDUs in Surrey and Victoria would attend a supervised injection facility if one were available.

## Conclusion

In summary, the supervised injection facility in Vancouver not only saves lives and reduces HIV and HCV transmission, but it is a life raft in a sea of misery for the people in the Downtown Eastside. The findings in this study are in keeping with more than 30 peer reviewed studies that show InSite has numerous positive provisions. Furthermore, this study’s qualitative data indicates that after years of operation, InSite has become a refugee camp for many of its users who escape death, violence and theft. In addition, InSite’s positive changes have contributed to a cultural transformation in drug use within the Downtown Eastside and neighboring communities. This study suggests there is an urgent need to expand InSite not only in the Downtown Eastside, but in other cities that have significant IDU populations. Opening more SIF in British Columbia could ultimately be a life raft in a sea of misery for the most vulnerable and marginalized people in our society.

## Endnotes

^a^Anecdotalism is defined as taking “lone entertaining instances” to be representative of a consistent theme [19].

^b^All quotes in this paper are verbatim to accurately reflect language usage by IDUs.

## Abbreviations

HCV: Hepatitis;HIV: Human Immunodeficiency Virus;SIF: Supervised injection facility;IDU: Injection drug user

## Competing interests

The authors declare that they have no competing interests.

## Authors’ contributions

All authors contributed to the design of this study. All authors read and approved the final manuscript.
